# A rapid real-time polymerase chain reaction-based live virus microneutralization assay for detection of neutralizing antibodies against SARS-CoV-2 in blood/serum

**DOI:** 10.1371/journal.pone.0259551

**Published:** 2021-12-10

**Authors:** Syed Hani Abidi, Kehkashan Imtiaz, Akbar Kanji, Shama Qaiser, Erum Khan, Kiran Iqbal, Marc Veldhoen, Kulsoom Ghias, J. Pedro Simas, Zahra Hasan

**Affiliations:** 1 Department of Biological and Biomedical Sciences, Aga Khan University, Karachi, Pakistan; 2 Department of Pathology and Laboratory Medicine, Aga Khan University, Karachi, Pakistan; 3 Instituto de Medicina Molecular João Lobo Antunes, Faculdade de Medicina, Universidade de Lisboa, Lisbon, Portugal; 4 Católica Biomedical Research; Católica Medical School, Universidade Católica Portuguesa, Lisboa, Portugal; Waseda University: Waseda Daigaku, JAPAN

## Abstract

**Background:**

Individuals recovering from COVID-19 are known to have antibodies against the Spike and other structural proteins. Antibodies against Spike have been shown to display viral neutralization. However, not all antibodies against Spike have neutralizing ability although they may be cross-reactive. There is a need for easy-to-use SARS-CoV-2 neutralizing assays for the determination of virus-neutralizing activity in sera of individuals. Here we describe a PCR-based micro‐neutralization assay that can be used to evaluate the viral neutralization titers of serum from SARS-CoV-2 infected individuals.

**Methods:**

The SARS-CoV-2 strain used was isolated from a nasopharyngeal specimen of a COVID-19 case. The limiting dilution method was used to obtain a 50% tissue culture infective dose (TCID50) of Vero cells. For the micro‐neutralization assay, 19 serum samples, with positive IgG titers against Spike Receptor-Binding Domain (RBD) were tested. After 24 hours, infected cells were inspected for the presence of a cytopathic effect, lysed and RNA RT-PCR conducted for SARS-CoV-2. PCR target Ct values were used to calculate percent neutralization/inhibition of SARS-CoV-2.

**Results:**

Out of 19 samples, 13 samples gave 100% neutralization at all dilutions, 1 sample showed neutralization at the first dilution, 4 samples showed neutralization at lower dilutions, while one sample did not demonstrate any neutralization. The RBD ODs and neutralization potential percentages were found to be positively correlated.

**Conclusion:**

We describe a rapid RT-PCR-based SARS-CoV-2 microneutralization assay for the detection of neutralizing antibodies. This can effectively be used to test the antiviral activity of serum antibodies for the investigation of both disease-driven and vaccine-induced responses.

## Introduction

The first cases of SARS-CoV-2 infection were reported in the city of Wuhan, China on December 1, 2019, as *pneumonia* with an unknown etiology [1, 2]. The first reported case outside the Chinese territory followed within months in Thailand and on March 11^th,^ 2020, SARS-CoV-2 was declared a *pandemic* [1, 2]. Globally, as of June 7, 2021, SARS-CoV-2 has infected 172 630 637 individuals, while 3 718 683 deaths have been recorded (https://www.who.int/emergencies/diseases/novel-coronavirus-2019).

SARS-CoV-2 is an RNA virus that uses the human angiotensin‐converting enzyme 2 (ACE2) receptor to infect host cells [3, 4]. Attachment to ACE2 and subsequent entry of SARS-CoV-2 is mediated by the Spike glycoprotein via the receptor-binding domain (RBD) [3, 4]. Spike protein is a target for antiviral antibodies, and the RBD domain, in particular, is the major focus for neutralizing antibodies [3–6]. Studies have shown that patients who recovered from COVID-19 or, those who are vaccinated, maintain a high antibody titer against the Spike/RBD protein [3–7]. However, individuals can get re-infected with SARS-CoV-2, suggesting that not all antibodies to S protein have the capacity to neutralize or are not long-lasting enough to give a durable response [8–10]. Focused studies on neutralizing antibodies in infected or vaccinated individuals are of significant value, as a correlation between antibody titers and virus neutralization is essential to measure the efficacy of the vaccination programs, especially against emerging variants [8, 11–13]. There are several live virus neutralization assays in use, where the most common one is based on a plaque reduction neutralization test [4]. These assays require an agarose overlay, which makes the assay laborious to perform. Other live-virus neutralization assays are ELISA-based which are more effective than the plaque assays but still involve antibody labeling and processing steps [4]. Pseudotype virus assays are an alternative to live virus assays however, these give an ‘approximation of the actual virus’ and may not represent the naturally circulating or newly emerging strains [4]. Here, we describe a neutralizing assay for SARS-CoV-2 using a real-time PCR-based assay output that can be completed within 24 hours and can effectively be used to test neutralization potential of antibodies against viruses including emerging antibody ‘escape’ variants.

## Materials and methods

### Sample collection

Serum was separated from blood taken from convalescent individuals after four weeks of their recovery from COVID-19. The samples were collected after obtaining informed consent from the patients. This study was approved by Aga Khan University, Ethical Review Committee (ERC# 2020-5152-11688).

### Cell culture, virus isolation and propagation

Vero cells (ATCC CCL-81) were cultured in T25cm^2^ flasks containing DMEM media supplemented with 10% Fetal Calf Serum (FCS), 1% L-glutamine 200mM, 1% penicillin G (100U/ml), streptomycin (100ug/ml) at 37°C and 5% CO^2^ until 80–90 confluency was achieved.

Nasopharyngeal swab (NPS) in viral transport medium from a PCR-positive SARS-CoV-2 case from June 2020, during the first wave of COVID-19 in Pakistan, was used for virus isolation. The particular viral isolate has not been sequenced but our data from that time period identified the G clade strains to be predominant in Pakistan (https://www.biorxiv.org/content/10.1101/2020.08.04.234153v1). Fifty microliters of serum-free DMEM were pipetted into columns 2–12 of a 96-well tissue culture plate; subsequently, 100μL of clinical specimens were pipetted into column 1 and serially diluted 2-fold across the plate (columns 2–12; from 1 log to 11 logs). Cultured Vero cells (80–90% confluent) were trypsinized and resuspended in DMEM containing 10% FCS and antibiotics at a concentration of 1×10^6^ cells/mL. A hundred microliter of cell suspension was directly added to the wells of the 96-well plate containing dilutions of the clinical specimen (NPS) and mixed gently by pipetting. Inoculated cultures were grown in a humidified incubator at 37°C with 5% CO^2^ for 4 days. The infected Vero cell line was observed daily for the presence of CPE using an inverted optical microscope (Olympus, Japan), and the virus was harvested when 80%‐90% of the cells manifested CPE. The end‐point titers were calculated according to the Reed & Muench method [14] based on eight replicates for titration. The culture medium was centrifuged at +4°C 1600rpm for 8 minutes, to remove the cell debris, and then aliquoted and stored at 80°C.

### Spike and RBD ELISA

Recombinant Spike and RBD proteins were kindly provided by Dr. Paula Alves, IBET, NOVA University, Portugal. We used ELISA to quantify serum antibodies against both SARS-CoV-2 Spike and RBD proteins. For this, a 96-well ELISA plate was coated with 50μl of Spike or RBD protein at a concentration of 2 μg/ml in PBS [15, 16]. The coated plate was stored at 4°C overnight. The next day, the wells were blocked with 200 μl of PBS + 0.1% Tween (PBS-T) + 3% non-fat milk, and the plate was incubated for 1 hour at room temperature. After incubation, wells were washed with washing buffer. Subsequently, the serum samples were diluted at 1:100 in PBS-T + 1% non-fat milk powder, and 100 μl of serum was added to the wells and the plate was incubated for 2 hours at room temperature. After incubation, the wells were washed with washing buffer and stained with secondary antibodies. For secondary staining, goat anti-human IgG Fc (HRP) was diluted 1:40,000 in PBS-T + 1% non-fat milk, and 50 μl was added to each well; the plate was incubated for 1 hour at room temperature. Following incubation, the plate was washed with washing buffer. Finally, for color development, 100 μl of TMB substrate solution was added to each well and the plate was incubated for 10 minutes. The reaction was stopped by adding 50 μl of 0.5M sulfuric acid to each well. The optical density of each well was read at 450nm immediately after adding the stop solution. A cut-off OD of 0.5 was used for both Spike and RBD proteins to detect positive samples.

### PCR-based microneutralization assay

For the assay development and optimization, we used five serum samples positive for anti-RBD antibodies as determined by ELISA. Patient serum samples were heat‐inactivated for 30 minutes at 56°C. Three ten-fold serum dilutions (1:10, 1:100, and 1:1000) were prepared in media. Each serum dilution was mixed with an equal volume of viral solution containing 100 TCID50 of SARS‐CoV‐2. The serum‐virus mixture was incubated for 1 hour at 37°C in a humidified atmosphere with 5% CO_2_. After incubation, 100ul of the mixture at each dilution was added in duplicates to a 96-well cell culture plate containing a semi-confluent Vero cell monolayer. The plates were incubated for 24hrs at 37°C in a humidified atmosphere with 5% CO_2_ [17]. Cells without virus served as ‘cell line control’, while cells with the virus without serum served as ‘virus control’.

After incubation of 96-well plates for 24 hours, the supernatant was carefully removed, and cells were washed with DMEM media. After the final wash, RNA was extracted from the cells using the Qiagen Viral Extraction Kit (cat no:52906; Qiagen-Germany) following the manufacturer’s instructions. The RNA was also used to perform a rapid real-time PCR using Novel Coronavirus (2019-nCOV) Nucleic Acid Diagnostic Kit (PCR Fluorescence Probing) of Sansure Biotech (S3102E) (Changsha, China) on CFX96™ Real-Time PCR thermal cycler (Bio-Rad Laboratories, Inc.). The assay sensitivity of Sansure Biotech is 1000 copies/ml [18]. This was validated in our laboratory to detect SARS-CoV-2 viral RNA with a Ct cut-off of 39. For PCR, 30 μL PCR-Master mix (including 2019-nCoV-PCR Mix and 2019-nCoV-PCREnzyme Mix, containing primers, probes, dNTPs, MgCl2, RNasin and PCR buffer for the 2019-nCoV-PCR Mix and RT enzyme and Taq enzyme for the 2019-nCoVPCR-Enzyme Mix) was added to a PCR reaction tube with 20 μL of the extracted RNA sample. The first two steps “reverse transcription” and “cDNA preparation” were performed at 50°C (30 min) and 95°C (1 min), each with a single cycle, respectively. For PCR amplification following conditions were used: 45 cycles, comprising of denaturation for 15-second at 95°C, and annealing for 30-second at 60°C, followed by cooling to 25°C for 10 seconds to finalize the process. The CFX96 in-built software was used for the calculation of the cycle threshold (Ct) values. For 2019-nCoV-PCR, a negative control was defined as Ct value >40, while positive control was defined as Ct value ≤ 35, as per kit’s instructions [19]. Alternative to the commercial kit, the qPCR was also performed using SARS-CoV-2 specific primers as described in S1 File.

The SARS-CoV-2 PCR Ct values obtained for each serum-virus well, and control wells containing cells alone and virus control, were averaged for each sample. The average Ct values obtained were used to measure the percent inhibition/neutralization using the formula [4]: 100 − ((*N*‐average Ct of ʻcell line control’ wells)/(average Ct of ʻvirus controlʼ wells‐average of ʻ cell line control’ wells)*100), where *N* is the average Ct for each well/sample.

Correlation between the RBD titers and neutralizing potential was determined using the Pearson correlation test using the GraphPad prism. P-value <0.05 was considered significant.

## Results

Nineteen different sera having positive antibodies for Spike, having OD450nm from 1.0 to 3.56, and RBD, having OD450nm from 0.89–1.1, were used in the neutralization assay (Table 1). Three ten-fold dilutions (1:10, 1:100, and 1:1000) of each serum sample were used in the SARS-CoV-2 PCR-based neutralization assay. The averaged Ct values for Vero cell line and SARS-CoV-2 virus controls were 0 and 33, respectively. The Ct values for antibody /serum samples were measured after 2-hour incubation with the virus.

**Table 1:** Optical density and Ct values for samples, and controls: Averaged OD (450nm) against SARS-CoV-2 spike and RBD protein are shown.

Samples	OD against Spike (450nm)	OD against RBD (450nm)	Averaged Ct values		
			1:10	1:100	1:1000
Sample 1	1.004	1.091	0	0	0
Sample 2	1.403	1.201	0	0	0
Sample 3	3.56	0.963	0	0	0
Sample 4	1.845	0.76	0	0	0
Sample 5	2.683	1.03	0	0	0
Sample 7	2.416	1	0	0	0
Sample 8	3.274	1.026	0	0	0
Sample 9	1.403	1.201	0	0	0
Sample 10	1.5	1.1	0	0	38.42
Sample 11	2.514	1.22	0	0	0
Sample 12	0.992	2.02	0	0	0
Sample 13	1.129	1.374	0	0	36.67
Sample 14	2.358	0.833	0	0	36.09
Sample 15	1.218	0.998	0	33.07	38.77
Sample 16	1.363	1.163	0	0	39.61
Sample 17	0.654	1.065	0	0	0
Sample 18	1.102	0.895	37.03	38.16	38.25
Sample 21	1.168	0.809	0	0	0
Sample 24	1.545	1.759	0	0	0
VC	-	-	33	33	33
CC	-	-	0	0	0

Ct values were obtained in the neutralization assay for 19 serum samples, cell line control, and virus control.

**Fig 1:**
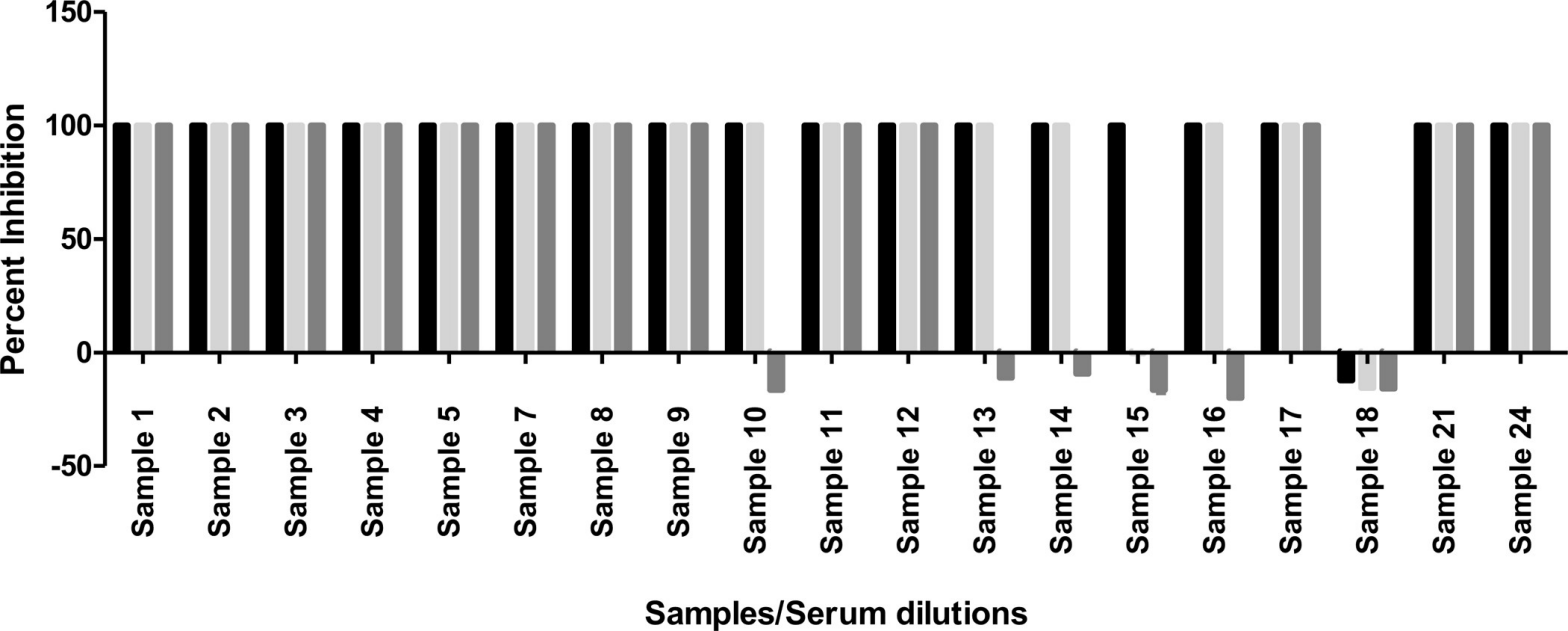
Percent inhibition/neutralization exhibited observed for each serum sample: Virus neutralization potential of the serum was tested at three different serum dilutions (1:10, 1:100, 1:1000). Error bars above each bar represent the standard error of the mean.

Incubation of these sera with the virus gave a result that was quantified by the PCR assay (Table 1). The Ct values obtained were used to calculate percent neutralization for the test samples. Out of 19 samples tested, 13 displayed virus inhibitions at all three dilutions, while four samples (10, 13, 14, 16) gave 100% inhibition at the first 2 dilutions only (Fig 1). Similarly, sample 15 gave 100% inhibition at first dilution (1:10), while sample 18 did not display any viral neutralization at the three dilutions tested (Fig 1). Overall, the RBD ODs and neutralization potential percentages were found to be positively correlated (R = 0.0385).

## Discussion

The availability of an assay that can detect the neutralization potential of the antibody that can correlate with antibody titer, such as those measured through ELISA, has significant utility for both seroepidemiological studies and also to measure the real-time impact of antibody titers in the context of vaccination programs, to determine the longevity of the vaccine-derived immunity in different age-groups [8–13]. The presence of neutralization antibody titers, induced either by natural infection or vaccination, is crucial for the prevention of possible reinfection and limiting the viral transmissions [8, 20]. Building on the work by Amanat *et* al. [4], we present a PCR-based neutralization assay that can provide rapid information about the presence of neutralizing antibodies in recovered or vaccinated individuals. The use of live viruses for the neutralization effect and the use of specific primers for detection of viral RNA makes this assay useful in testing the presence of neutralization antibodies not only against wild type but also new and emerging variants. Similarly, the use of real-time PCR-based detection increases the sensitivity of the assay as low copies of the virus can also be detected. Any real-time PCR assay for SARS-CoV-2 RNA may be used as a read-out for Ct values to determine the viral load of the assay. This may be in the form of a commercial kit as used here or alternately, an in-house using primers such as those provided in the S1 File may be employed.

In our assay, 19 samples were tested, out of which 13 displayed 100% virus inhibition at all dilutions tested while four showed inhibitions at low dilutions only. One sample did not exhibit any virus neutralization potential. The correlation between optical density reading of IgG antibodies to RBD OD and their neutralization potential was found to be positively correlated. This observation is in agreement with several studies that have shown a positive correlation between RBD titers and viral neutralization [4, 21–25]. On the contrary, the samples exhibiting failure to neutralize at a lower dilution may suggest that the antibodies present in the serum were cross-reactive to RBD but did not have neutralizing capacity. These findings are not unprecedented as one study exploring the correlation between ELISA titers and neutralization potential found neutralizing antibodies in one-third of the individuals with positive results on immune-detection assays [12]. This suggests that the quality of the antibodies (capacity of the antibody to neutralize the virus) rather than the IgG titers is more important to evaluate the protective response against infection, thus, justifying the use of neutralizing assays in the seroepidemiological studies. However, these results need to be interpreted with caution as the decline/absence of neutralizing antibodies does not mean an absence of protective immunity against disease, as protection can be conferred by other cells, such as T cells [12].

Our study has some limitations which include the relatively small number of samples used, also that we only selected specimens with high RBD/Spike antibody titers. Further, this assay requires work to be conducted in a BSL-3 facility. However, the value of this assay is that it is easy to interpret and can be used to investigate neutralizing potential of antibodies against SARS-CoV-2 using any diagnostic RT-PCR assay for the virus. We used three-point dilutions to measure the neutralization potential of serum samples. Several studies evaluating serum antibody responses have used three-point dilutions (1:10, 1:100, and 1:1000) to evaluate the serum inhibitory activity, as used in this study [26–28]. Other studies have extended the dilution to 1:1000 [27]. We observed either no inhibition at three dilutions, and in this case, further inhibition at higher dilutions is also unlikely; or inhibition at 1:100 and/or 1:1000, where a greater number of titrations may have given a cut-off point for serum inhibitory activity. This was however not the scope of the current study.

A major advantage of this assay is that because it is not virus-specific such as a pseudotyped assay would be [4], it would be possible to use the same to test the neutralizing potential of test sera against viruses of any required lineage and in particular, could be used to test against particular SARS-CoV-2 variants of concern.

## Conclusions

In summary, we present a simple PCR-based SARS-CoV-2 assay that can detect the presence of neutralizing antibodies in serum samples. The assay can also be used to determine the virus neutralization potential of serum from vaccinated or recovered individuals and/or determine the emergence of vaccine escape infections.

## Supporting information

S1 File Details of primers and protocol for alternate PCR strategy. (DOCX)
