# Supplementary material for: A rapid real-time polymerase chain reaction-based live virus microneutralization assay for detection of neutralizing antibodies against SARS-CoV-2 in blood/serum
Source: PLoS One. 2021 Dec 10;16(12):e0259551. doi: 10.1371/journal.pone.0259551 (PMC8664206; doi:10.1371/journal.pone.0259551)
Supplement: S1 File — (DOCX) [file pone.0259551.s001.docx]

**SARS-CoV-2 qPCR amplification using virus-specific primers:**

For primer-based qPCR assay following primers were used: Rd RNA pol: For:AGAATAGAGCTCGCACCGTA and Rev:CTCCTCTAGTGGCGGCTATT; S- Antigen: For:GCTGGTGCTGCAGCTTATTA and Rev:AGGGTCAAGTGCACAGTCTA; N-Antigen: For: CAATGCTGCAATCGTGCTAC and Rev:GTTGCGACTACGTGATGAGG; E- Antigen: For:TTCGGAAGAGACAGGTACGTTA and Rev:AGCAGTACGCACACAATCG, GAPDH: For:CAATGACCCCTTCATTGACC and Rev: TTGATTTTGGAGGGATCTCG. For primer-based amplification, the thermocycling conditions are as follows: 50 °C for 2 min, 95 °C for 10 min, 40 cycles of 95 °C for 15 s, and 62 °C for 1 min, and by a melting curve stage of 95 °C for 10 s and 60 °C for 1 min.
